# SMRT sequencing analysis reveals the full-length transcripts and alternative splicing patterns in *Ananas comosus* var. *bracteatus*

**DOI:** 10.7717/peerj.7062

**Published:** 2019-06-21

**Authors:** Jun Ma, Yixuan Xiang, Yingyuan Xiong, Zhen Lin, Yanbin Xue, Meiqin Mao, Lingxia Sun, Yujue Zhou, Xi Li, Zhuo Huang

**Affiliations:** College of Landscape Architecture, Sichuan Agricultural University, Chengdu, Sichuan, China

**Keywords:** Full-length transcriptome sequence, Alternative splicing, *Ananas comosus* var. *bracteatus*, Albino, SMRT sequencing, Chimeric

## Abstract

**Background:**

*Ananas comosus* var. * bracteatus* is an herbaceous perennial monocot cultivated as an ornamental plant for its chimeric leaves. Because of its genomic complexity, and because no genomic information is available in the public GenBank database, the complete structure of the mRNA transcript is unclear and there are limited molecular mechanism studies for *Ananas comosus* var. *bracteatus*.

**Methods:**

Three size fractionated full-length cDNA libraries (1–2 kb, 2–3 kb, and 3–6 kb) were constructed and subsequently sequenced in five single-molecule real-time (SMRT) cells (2 cells, 2 cells, and 1 cell, respectively).

**Results:**

In total, 19,838 transcripts were identified for alternative splicing (AS) analysis. Among them, 19,185 (96.7%) transcripts were functionally annotated. A total of 9,921 genes were identified by mapping the non-redundant isoforms to the reference genome. A total of 10,649 AS events were identified, the majority of which were intron retention events. The alternatively spliced genes had functions in the basic metabolism processes of the plant such as carbon metabolism, amino acid biosynthesis, and glycolysis. Fourteen genes related to chlorophyll biosynthesis were identified as having AS events. The distribution of the splicing sites and the percentage of conventional and non-canonical AS sites of the genes categorized in pathways related to the albino leaf phenotype (ko00860, ko00195, ko00196, and ko00710) varied greatly. The present results showed that there were 8,316 genes carrying at least one poly (A) site, which generated 21,873 poly (A) sites. These findings indicated that the quality of the gene structure and functional information of the obtained genome was greatly improved, which may facilitate further genetic study of *Ananas comosus* var. * bracteatus*.

## Introduction

*Ananas comosus* var. *bracteatus* (red pineapple) is an herbaceous perennial monocot originating from South America, and it belongs to the family Bromeliaceae, genus Ananas, and species *A. comosus* (L.) *Merr*. (diploid, 2*n* = 2*x* = 50) (Bartholomew, Paull & Rohrbach, 2003). Most plants of this family are commercially cultivated for their delicious fruit, high-quality fibre of their stem and leaves (Collins, 1960; Montinola, 1991) or their rich secondary metabolites (Taussig & Batkin, 1998; Wen, Wrolstad & Hsu, 1999; Takata & Scheuer, 1976; Rocha & Kaplan, 2000; Bartholomew, Paull & Rohrbach, 2002; Beauman, 2006). However, *A. comosus* var. *bracteatus* is cultivated commercially as an important ornamental plant for its colourful chimeric leaves and red fruit. A chimeric leaf can be used as a marker in breeding (Burge, Morgan & Seelye, 2002), and it is an optimal material for the study of plant tissue and organ formation and development (Satina, Blackeslee & Avery, 1940; Stewart, Semeniuk & Dermen, 1974) as well as the interaction between cells (Stegemann & Bock, 2009). Limited genomic information is available. Ming et al. (2015) published information for the CB5 DNAseq library (SRR5963871), and transcriptomic data was published by Li et al. (2017) (Bioproject PRJNA389361) and Ma et al. (2015) (SRX681749). Because of its genomic complexity and limited genomic information in the public GenBank database, studies on the molecular mechanism involved in the growth and development of this plant are limited. Therefore, high-throughput transcriptome sequencing was performed by our laboratory to generate large quantities of transcript sequences (Ma et al., 2015; Li et al., 2017).

Next generation sequencing technologies have short read lengths that are not capable of spanning entire transcripts (Koren et al., 2012), and it is difficult to predict gene structures correctly with the current prediction programs using short transcript sequencing reads (Coghlan et al., 2008). Large-scale sequencing of cDNA is an effective method for gene discovery and genome annotation (Wang et al., 2016). Expressed sequence tag (EST) sequences and transcriptome sequences rarely cover entire transcripts (Xu et al., 2015). Traditional RNA-seq analysis remains affected by substantial difficulties with isoform identification and quantification (Ning et al., 2017). In contrast, assembled full-length cDNAs are the gold standard for annotation, but they can be obtained for only relatively small numbers of genes and at considerable cost (Wang et al., 2016). Full-length cDNA sequences are fundamental resources to study structural, functional, and comparative genomics (Luo et al., 2017). Single-molecule real-time (SMRT) sequencing overcomes the limitation of short read lengths by enabling the generation of kilobase-sized sequencing reads (Sharon et al., 2013). The present study performed full-length sequencing of the transcriptome of *A. comosus* var. *bracteatus* to improve the overall accuracy of gene prediction in non-model species without a high-quality reference genome.

## Materials & Methods

### Plant materials and sample preparation

Leaves, stems, and roots were collected from 3-year-old chimeric plants of *A. comosus* var. *bracteatus* grown at the experimental nursery of Sichuan Agricultural University. Complete green shoots, complete white shoots, and calluses were collected from plants derived via tissue culture (Li et al., 2017). Tissues were immediately frozen in liquid nitrogen. For each tissue, at least five plants were pooled. Total RNA was prepared with TRIzol reagent (Invitrogen) following the protocol provided by the manufacturer. Isolated RNA was quantified and qualified by NanoDrop and Agilent 2100 Bioanalyzer instruments.

### PacBio library construction and sequencing

RNAs of each tissue sample type were pooled into an equal concentration and then used for size selection (1–2 kb, 2–3 kb, and 3–6 kb). An Isoform-Sequencing (Iso-Seq) library was constructed for each size fraction based on the Iso-Seq protocol. cDNA amplification was conducted through BluePippin (Sage Science) size selection criterion. SMRTbell libraries were prepared using the Pacific Biosciences DNA Template Prep Kit 2.0. Genome sequencing was performed using a PacBio RS II instrument. The high-throughput sequencing reported in the present study was performed by Biomarker Technology Co. (Beijing, China).

### Error correction of PacBio reads

The size fraction within each tissue in this experiment was individually run through the Iso-Seq pipeline contained in the SMRT Analysis software package. Reads of inserts (ROIs) were generated based on the method described by https://github.com/PacificBiosciences/cDNA_primer/wiki/Understanding-PacBio-transcriptome-data#readexplained. ROIs shorter than 50 bp in length were discarded. The pipeline then classified the ROIs as full-length non-chimeric or non-full-length reads according to whether 5′/3′ cDNA primers and a poly (A) tail were simultaneously observed. Consensus isoforms were identified by iterative algorithmic clustering for error correction and further data cleaning. The PacBio SMRT reads generated in the present study have been submitted to the BioProject database of National Centre for Biotechnology Information (accession number PRJNA494788).

### Transcript function annotation

Transcripts were compared against NR (NCBI non-redundant protein sequences), S Swiss-Prot (a manually annotated and reviewed protein sequence database), and KOG/COG/eggNOG (Clusters of Orthologous Groups of proteins) databases by BLASTX v2.2.31 (cut-off *E*-value ≤1^*e*−5^). Gene Ontology (GO) was annotated using Blast2GO v2.5 based on NR annotation. Kyoto Encyclopedia of Genes and Genomes (KEGG) pathways were annotated by KOBAS v2.0. HMMER v3.1b2 was used to compare amino acid sequence transcripts against the Protein family (Pfam) database for Pfam annotation.

### Predictions of open reading frames (ORFs), simple sequence repeats (SSRs), and long non-coding RNAs (lncRNAs)

The ORFs in transcripts were predicted by the TransDecoder v2.0.1 package (https://transdecoder.github.io/). Full-length transcripts were designated as those transcripts containing complete ORFs and 5′- and 3′-untranslated regions (UTRs). Putative SSRs were identified using MISA (MIcroSAtellite identification tool; http://pgrc.ipk-gatersleben.de/misa). Only transcripts of ≥500 bp in length were included in SSR detection (Luo et al., 2017). The PLEK (https://sourceforge.net/projects/plek) alignment-free tool was used to predict lncRNAs.

### Poly (A) site identification

Poly (A) site identification from the coding region of genes in the present experiment was based on the method published by Abdel-Ghany et al. (2016) with minor modifications. Transcripts were classified into three types as follows: 5′-UTR, 3′- UTR or internal, corresponding to whether the site occurred upstream of the start codon, downstream of the stop codon or in between the start and stop codon, respectively. Aligned reads were selected for poly (A) events using a previously published method (Abdel-Ghany et al., 2016).

### Alternative splicing (AS) analysis

The redundancy removed transcripts were used for AS analysis. AS events including, IR, ES, AD, AA and MEE, were identified by the AStalavista tool using the default parameter for known and new transcripts (Foissac & Sammeth, 2007).

## Results and Discussion

### General properties of Iso-Seq data

**Figure 1:**
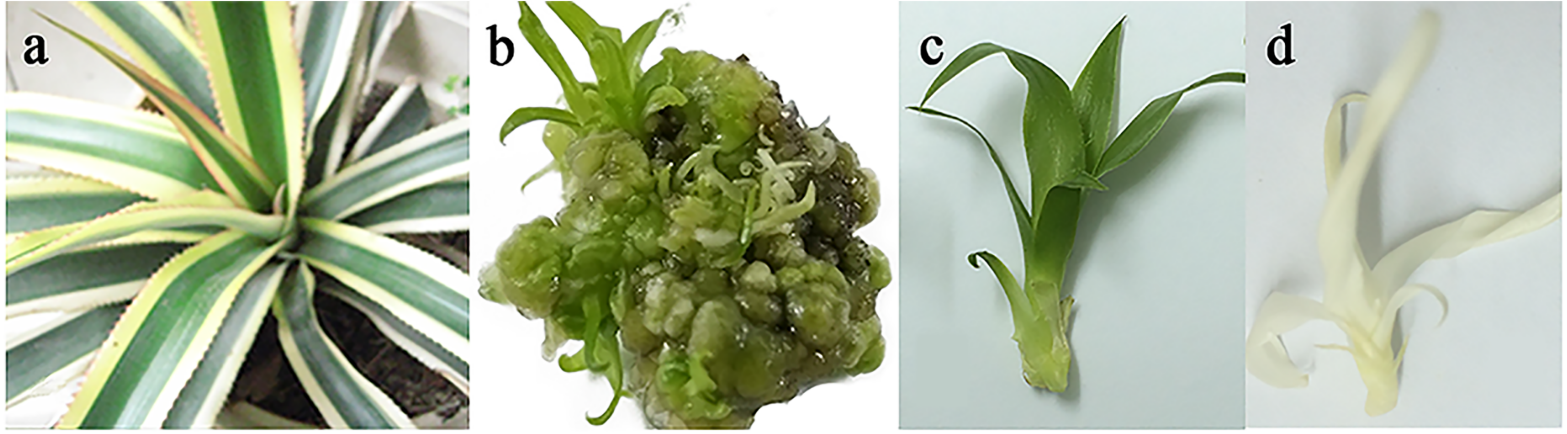
Plant materials used in this study. (A) Chimeric plant of *Ananas como sus* var. *bracteatus*. (B) Callus derived from stem of chimeric explant via tissue culture. (C) Complete green plant derived via tissue culture. (D) Complete white plant derived via tissue culture.

Because of the short-read length of Illumina data, it is difficult to predict gene structure and correctly annotate gene function (Coghlan et al., 2008). To address the limitation of the Illumina HiSeq platform, the transcriptome of *A. comosus* var. *bracteatus* was sequenced through the Pacific Biosciences Iso-Seq system. This platform can deliver long reads that enable precise construction of full-length splice variants. The leaves, stems and roots of chimeric plants as well as calluses, complete green shoots and complete white shoots derived via tissue culture were used for full-length transcriptome sequencing in the present study (Fig. 1). Size-fractionated full-length cDNA libraries (1–2 kb, 2–3 kb, and 3–6 kb) were constructed and subsequently sequenced in five SMRT cells (2 cells, 2 cells, and 1 cell, respectively). The sequences that met the most permissive criterion (minimum number of full passes = 0; and minimum sequence accuracy = 0.75) were filtered. In total, 304,215 ROIs were collected from five SMRT cells. The length distribution of these short-filtered ROIs is shown in Fig. S1A.

**Table 1:** General property of zero-full-pass ROIs.

cDNA Size	SMRT cell	Number of ROIs	Mean read length of insert	Mean read quality of insert	Number of full-length non-chimeric reads	Average full-length non-chimeric read length	Percentage of full-length reads (%)	Artificial concatemers (%)
1–2 kb	2	135,698	2,274	0.92	59,255	1,540	43.82%	0.35
2–3 kb	2	107,633	2,698	0.90	45,774	2,330	42.71%	0.42
3–6 kb	1	60,884	3,531	0.90	27,976	3,489	45.99%	0.09

Full-length reads were classified based on the presence of barcoded primers and poly (A) tails (Wang et al., 2016). A total of 133,005 full-length non-chimeric reads (flncROIs) were identified (Table 1). On average, 44.17% of all ROIs were full-length reads. The length distribution of the flncROIs is shown in Fig. S1B. The length distribution of flncROIs was consistent with the cDNA size distribution. The artificial concatemers represented 0.32% of the reads.

After Iterative Clustering for Error Correction (ICE) analysis using SMRT Analysis (v2.3.0) software, 55,010 consensus isoforms were identified. Among them, 42,182 consensus isoforms were high-quality (HQ) isoforms (accuracy ratio >99%). The statistical results of HQ and low-quality (LQ; accuracy ratio ≤99%) transcripts obtained from libraries of each size fraction are shown in Table 2. The length distribution of consensus isoform sequences obtained from different cDNA lengths is shown in Fig. S1C.

**Figure 2:**
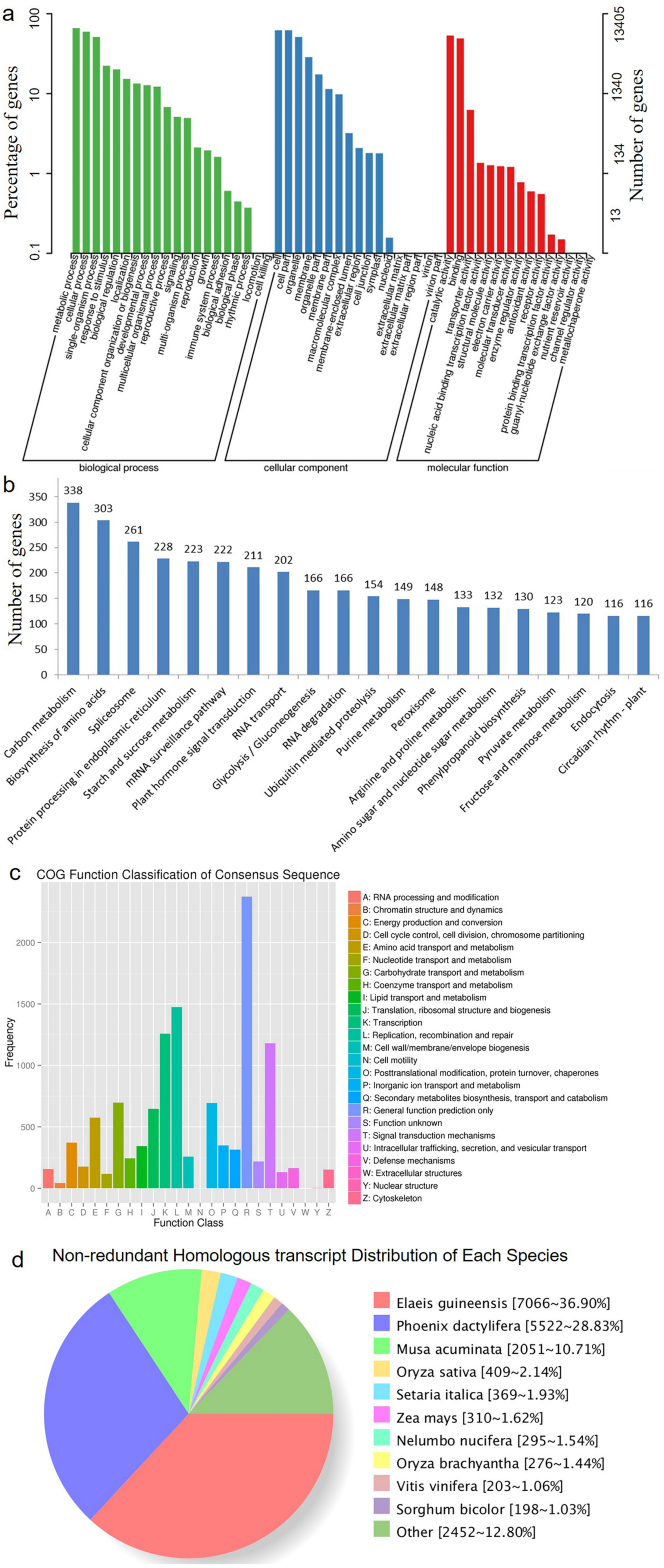
The annotation results of the identified transcripts. (A) The GO function annotation of the consensus sequences. (B) The KEGG function annotation of the consensus sequences. (C) The COG function classification of the consensus sequences. (D) The Nr homologous species distribution of the consensus sequences.

HQ transcript sequences were mapped to the *Acomosus* _321_v3 genome (Ming et al., 2015; https://phytozome.jgi.doe.gov/pz/portal.html#!info?alias=Org_Acomosus_er) using GMAP (Wu & Watanabe, 2005). To filter out potential truncated transcripts due to incomplete reverse transcription, reads differing only at the 5′-start site within the first exon were counted as redundant, and only the longest version was retained. ToFu analysis (https://github.com/PacificBiosciences/cDNA_primer/wiki/What-is-pbtranscript-tofu%3F-Do-I-need-it%3F) yielded 23,515 non-redundant isoforms, and these isoforms were compared to genomic transcripts using Cuffcompare (Trapnell et al., 2012). In total, 19,838 isoforms were identified as transcripts based on the compared result, and others were identical to known transcripts (transcripts published for Ananas comosus F153 genome). These transcripts greatly enriched the sequence database of *A. comosus* var. *bracteatus*. Among them, 19,185 (96.7%) transcripts were functionally annotated. The functional annotations of these transcripts are listed in Table 3 and Fig. 2. The largest category was biological processes followed by cellular component and molecular function in GO annotation (Fig. 2A). These transcripts were annotated to 123 KEGG pathways. The top five most annotated KEGG pathways were carbon metabolism, biosynthesis of amino acids, spliceosome, protein processing in endoplasmic reticulum, and starch and sucrose metabolism (Fig. 2B). The Clusters of Orthologous Groups (COGs) functional classification of the transcripts is shown in Fig. 2C. The non-redundant (NR) homologous analysis revealed that 36.9% of the transcripts were homologous to genes in *Elaeis guineensis*, 28.83% to *Phoenix dactylifera* and 10.71% to *Musa acuminate* (Fig. 2D). In total, 22,904 isoforms were mapped to known gene intervals, and 9,921 genes were derived. These isoforms were classified by Match Anno software (https://github.com/TomSkelly/MatchAnnot) as follows: 2,952 isoforms had a score of 5, and 745 of these had a splice termination motif; 1,670 isoforms had a score of 3, and 409 of these had a splice termination motif; 10,010 isoforms had a score of 2, and 2,342 of these had a splice termination motif; and 8,272 isoforms had a score of 0, and 1,844 of these had a splice termination motif. There were 588 isoforms that exhibited no homology to any annotated gene and were identified as new gene isoforms. The unique isoforms were compared to the reference genome to build the CIRCOS visualization of different data at the genome-wide level (Fig. S2).

**Table 2:** Statistics of iterative clustering for error correction analysis in each size fraction.

Size	Number of consensus isoforms	Average consensus isoforms read length	Number of polished high-quality isoforms	Number of polished low-quality isoforms	Percent of polished high-quality isoforms (%)
0 to 1 kb	357	767	313	44	87.68%
1 to 2 kb	21,445	1,526	18,294	3,151	85.31%
2 to 3 kb	18,616	2,379	14,218	4,397	76.38%
3 to 6 kb	14,059	3,563	9,346	4,713	66.48%
>6 kb	533	9,139	11	522	2.06%

**Table 3:** Functional annotation of *Ananas comosus* var. *bracteatus* identified in this study.

Database	Annotated_Number	300≤ length< 1000	length≥ 1000
COG_Annotation	8,060	26	8,034
GO_Annotation	13,405	55	13,350
KEGG_Annotation	8,871	38	8,833
KOG_Annotation	13,182	41	13,141
Pfam_Annotation	21,299	59	21,240
Swissprot_Annotation	13,928	54	13,874
eggNOG_Annotation	19,061	72	18,989
nr_Annotation	19,152	73	19,079
All_Annotated	19,185	76	19,109

### Open reading frame and simple sequence repeat prediction

In total, 18,156 ORFs were predicted from the 19,838 transcripts, and their length distributions were analysed (Fig. S3). Those transcripts containing complete coding sequences (CDSs) as well as an initiation codon, termination codon, 5′-UTRs and 3′-UTRs were defined as full-length transcripts. A total of 13,930 full-length transcripts were identified.

SSR markers are among the most widely used molecular markers in numerous organisms. Using the MIcroSAtellite identification tool, 25,971 SSRs were identified, and 4,573 of these SSRs were present in compound form. Most of the SSRs were mono-, di-, or tri-nucleotide repeats (Table S1). The SSR densities of different types of SSRs are listed in Fig. S4. The highest SSR density was detected for p1 SSR.

**Figure 3:**
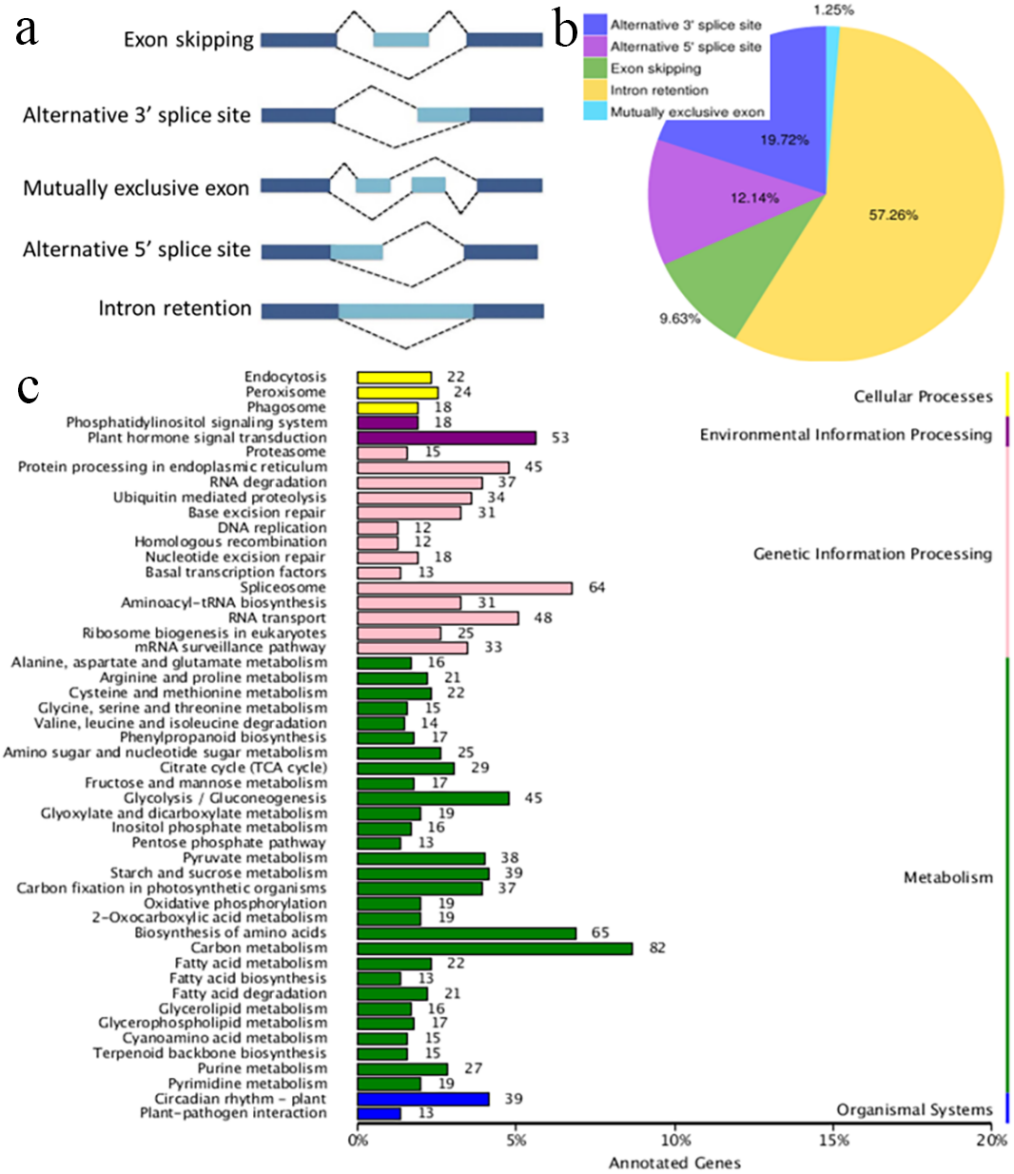
Characterization of alternative splicing events. (A) Schematic representation of five alternative splicing modes. (B) Distribution of different types of alternative splicing events. (C) The KEGG annotation of the alternatively spliced genes.

### Splice isoforms and AS

AS occurs by rearranging the pattern of intron and exon elements that are joined by splicing to alter the mRNA coding sequence (Braun et al., 2018). AS is a process that enables messenger RNA (mRNA) to direct synthesis of different protein variants (isoforms) that may have different cellular functions or properties (Kalsotra & Cooper, 2011; Zhou, Noushin & Adams, 2011; Wang et al., 2016). AS is a major cellular mechanism generating transcriptome plasticity and proteome diversity in plants (Reddy, 2007). The number of alternatively spliced isoforms in Ananas is still unknown. SMRT long reads can detect isoforms effectively and have been used to reveal the corresponding AS events in many organisms with an available reference genome (Au et al., 2013; Chen et al., 2014). Five main modes of AS (intron retention, exon skipping, alternative 3′ acceptor, alternative 5′ donor, and mutually exclusive exons; Fig. 3A) were ascertained using Astalavista software (Foissac & Sammeth, 2007). In the present study, a total of 10,649 AS events were identified, and this number was similar to that of pineapple (10,348 AS events; Wai et al., 2016a; Wai et al., 2016b). The distribution of AS events is shown in Fig. 3B. The majority of AS events were intron retention events (57.26%) in the present study, and 61.9% of the AS events have been reported to be intron retention events in pineapple (Wai et al., 2016a; Wai et al., 2016b). Intron retention can introduce stop codons, thereby activating nonsense-mediated decay (Wong et al., 2013), but it can also change ORFs, leading to functionally different variants (Wang et al., 2016). Alternative 3′-splicing (19.72%) was the second most prevalent (19%) mode, whereas mutually exclusive exons (1.25%) were the least frequent (Fig. 3B). Splicing mode distribution is not uniform across species and tissues (Wang et al., 2016). Moreover, multiple splicing modes can operate on a single transcript, potentially combinatorially generating diverse isoforms from a single gene. For example, the combination of alternative 5′ and 3′ splice sites along with intron retention and exon skipping yielded 51 observed isoforms of PacBiogene PB.685. The KEGG annotation of the AS genes is shown in Fig. 3C. The alternatively spliced genes functioned in the basic metabolism processes of plants, such as carbon metabolism, amino acid biosynthesis and glycolysis. The alternatively spliced genes were also annotated in plant hormone signal transduction pathways, which regulate environmental information processing. AS events were also found in genetic information processing which is important in plant growth and development.

**Figure 4:**
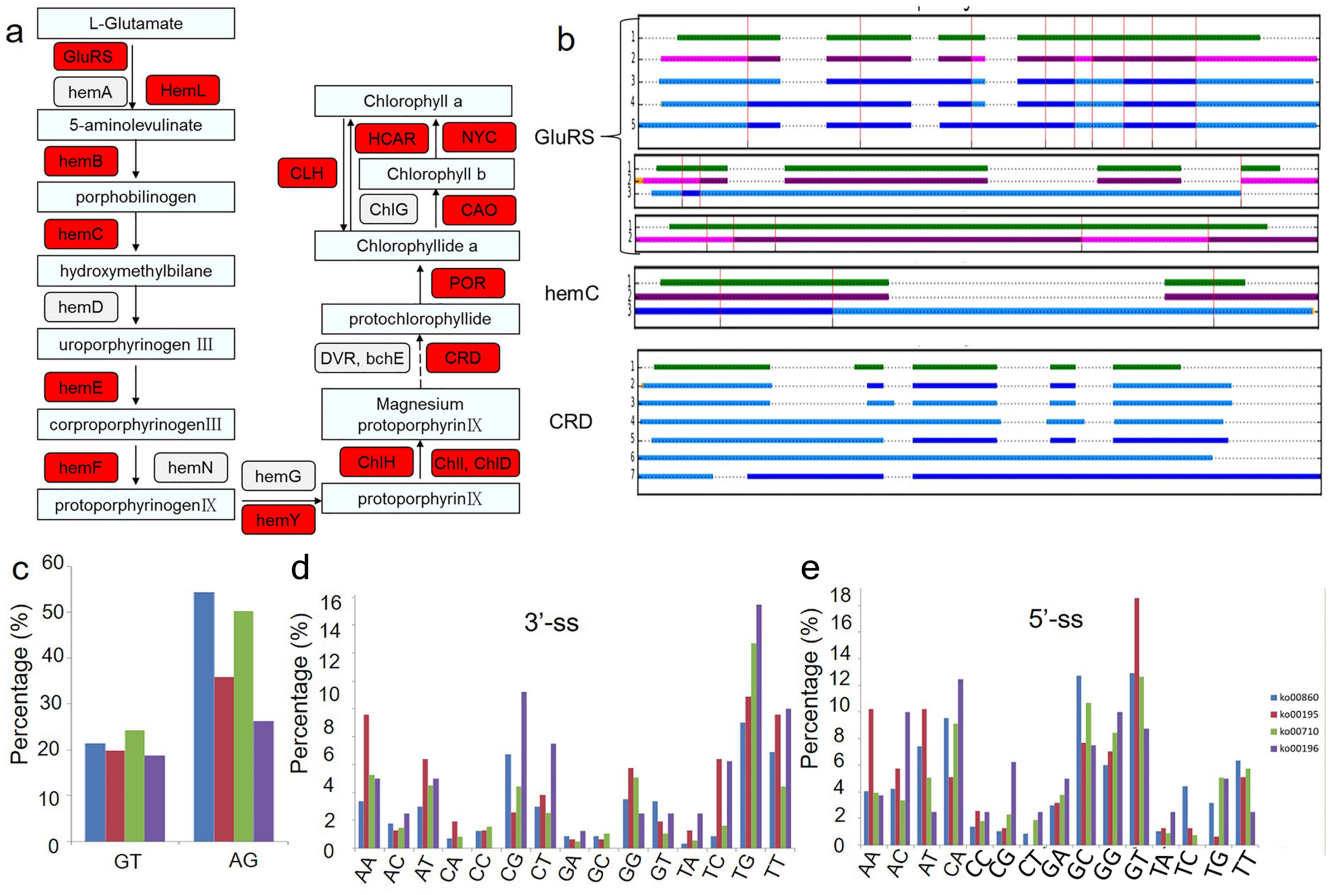
Alternative splicing events in transcripts involved in chlorophyll biosynthesis pathway. (A) Schematic diagram of the gene functions in chlorophyll biosynthesis. The genes identified to have AS events are marked in red colour. (B) Examples of alternative splicing in transcripts of genes related to chlorophyll biosynthesis. green indicates transcripts form annotation file , blue indicates transcripts from clusters and QScore ≥20 and IsoSeq exons match annotation exons one-for-one, DodgerBlue indicates transcripts from clusters and QScore < 20 and IsoSeq exons match annotation exons one-for-one, purple indicates transcripts from clusters and QScore ≥20 but IsoSeq exons match annotation exons not one-for-one, Magenta indicates transcripts from clusters and QScore < 20 but IsoSeq exons match annotation exons not one-for-one. (C) Frequency of the splicing sites (ss) selection between four KEGG pathways related to the albino leaf phenotype (ko00860, ko00195, ko00196, ko00710). GluRs: Glutamate–tRNA ligase; hemA: Glutamyl-tRNA reductase; hemL: Glutamate-1-semialdehyde 2,1-aminomutase; hemB: Delta-aminolevulinic acid dehydratase; hemC: Porphobilinogen deaminase; hemD: Uroporphyrinogen-III synthase; hemE: Heme oxygenase; hemF: Coproporphyrinogen-III oxidase; hemN: Coproporphyrinogen-III oxidase; hemG: Protoporphyrinogen oxidase; hemY: Protoporphyrinogen oxidase; ChlH: Magnesium-chelatase; ChlI: Magnesium-chelatase; ChlD: Magnesium-chelatase; DVR: Divinyl chlorophyllide a 8-vinyl-reductase; bchE: Carboxylic ester hydrolase; CRD: Mg-protoporphyrin IX monomethyl ester oxidative cyclase; POR: Protochlorophyllide reductase; CAO: Chlorophyllide a oxygenase; ChlG: Chlorophyll synthase; CLH: Chlorophyllase; HCAR: 7-hydroxymethyl chlorophyll a reductase; NYC: 7-hydroxymethyl chlorophyll a reductase.

Because chlorophyll biosynthesis is important for the formation of chimeric leaves, the AS events in the chlorophyll biosynthesis pathway were analysed (Fig. 4). Among the 21 genes related to the main chlorophyll biosynthesis pathway, 14 genes were identified as alternatively spliced (Fig. 4A). The gene structures and intron phases of glutamate-tRNA ligase (GluRs), hydroxymethylbilane synthase (hemC) and Mg-protoporphyrin IX monomethyl ester oxidative cyclase (CRD) are shown in Fig. 4B. This picture was produced by clusterView in a program called MatchAnnot (https://github.com/TomSkelly/MatchAnnot). clusterView produces a plot showing how the exons in a collection of transcripts match up. Transcripts can come from Iso-Seq clusters and/or annotation data.

In addition, the conservation of splicing sites in genes in four KEGG pathways related to the albino leaf phenotype (ko00860, ko00195, ko00196, and ko00710) were analysed (Figs. 4C, 4D and 4E). The distribution of the splicing sites across the four KEGG pathways varied greatly. Conventional splicing sites (5′-GT-AG-3′) accounted for approximately 45 to 75% of AS sites from each pathway, which differed from the pattern observed in *Arabidopsis* (Will & Luhrmann, 2011). The percentage of other non-canonical AS sites also varied in the four pathways, suggesting that a portion of differentially expressed AS events in different aspects of plant metabolism may result from the increased usage of the non-canonical splicing sites.

### Alternative polyadenylation

Alternative polyadenylation (APA) can generate transcript 3′-UTRs that contain different cis-regulatory elements, and this post-transcriptional regulation can lead to altered function, stability and translation efficiency of most target RNAs (Elkon, Ugalde & Agami, 2013; Bentley, 2014). Recent studies have revealed that APA improves transcriptome diversity through producing distinct transcript isoforms (Sherstnev et al., 2012; Wu et al., 2011; Shen et al., 2011). Differential polyadenylation in mRNAs plays a crucial modification role during plant development (Simpson et al., 2003; Liu et al., 2013). Conventional RNA-Seq has been extensively used in large genomics projects. However, it is impossible to quantify APA due to the missing poly (A) tails in RNA-Seq reads.

**Figure 5:**
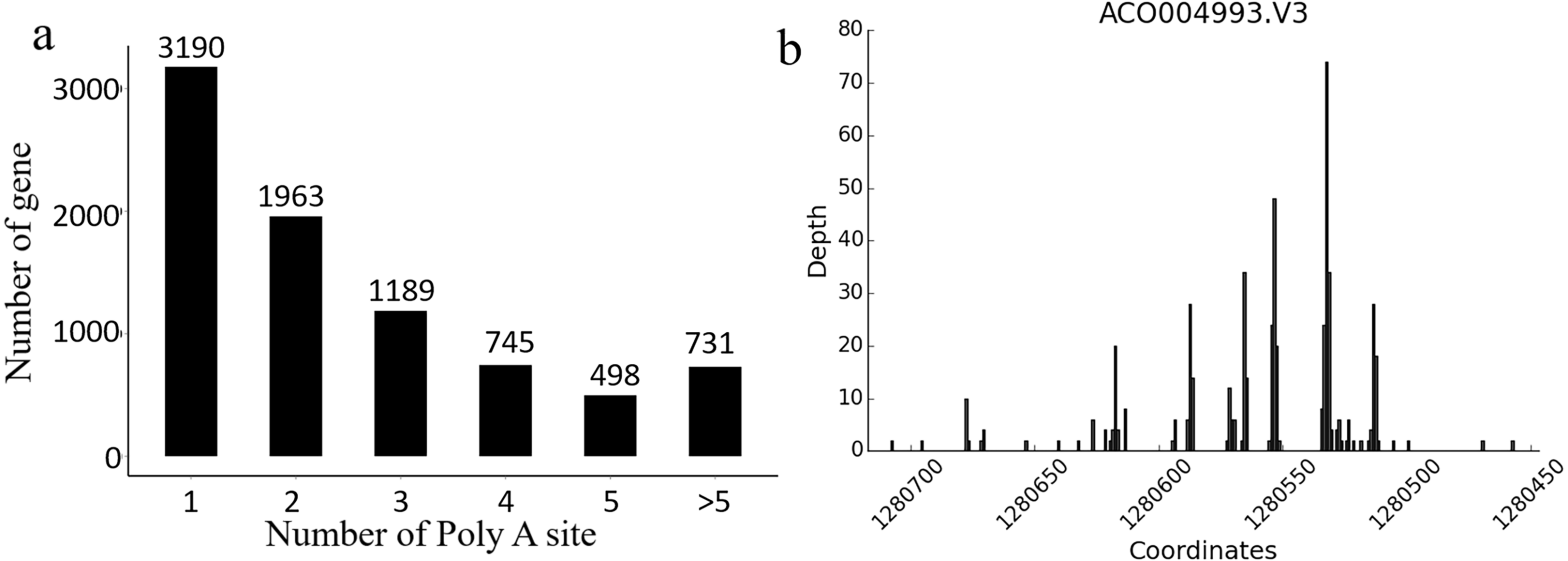
Alternative polyadenylation analysis. (A) Distribution of the number of poly(A) sites per gene. Poly(A) reads were clustered such that each site must have at least 2 reads supporting it, and no two clusters lie within 15 nucleotides of each other. (B) An example of a gene that produces transcripts with multiple polyadenylation sites. Distribution of the number of poly(A) reads is shown on the *y*-axis, and the estimated cluster centres are shown as vertical lines on the *x*-axis.

In *Ananas comosus* var. *bracteatus*, the transcriptome complexity resulting from APA events is still unknown. The present results showed that there were 8,316 genes carrying at least one poly (A) site, and these genes generated 21,873 poly (A) sites in total. There were 5,126 genes containing no less than two APA sites (Fig. 5A). Of note, 24.8% genes with more than one alternative poly (A) site occupied a preferred site, which was more than 50% of the poly (A) reads for that gene alignment to a single poly (A) site. These results also indicated that there was a total of 124 poly (A) sites identified in the CDS regions among 100 genes, and 44 poly (A) sites were found in the 5′-UTRs among 20 genes. These findings also supported the accuracy and reliability Iso-Seq technology (Fig. 5B). Together, these results implicated that APA may be a usual event in *Ananas comosus* var. *bracteatus* as it is in sorghum (Abdel-Ghany et al., 2016).

### Transcription factor (TF) isoforms produce functional variants

TFs are key components involved in the transcriptional regulatory system. Using the present data, 1,094 TF isoforms were identified, and 907 of these were novel isoforms. Compared to the *Acomosus* _321_v3 genome, 55 novel TFs were identified in the present data by iTAK. The increase in TF isoforms was broadly distributed (54 of the 58 TF families), and several families had a particularly high prevalence of isoforms. For example, the present data revealed 71 new isoforms of the C3H TF, nearly tripling the number of annotated variants. C3H proteins are zinc finger proteins, which are members of a large family of transcription regulators that modulate the expression of downstream stress responsive genes in plants (Kodaira et al., 2011). These novel isoforms will provide additional mechanistic insights into plant stress response. A noteworthy expansion in the number of transcript isoforms was also observed for the bHLH, GARP-G2-like and FAR1 TF families.

### LncRNA identification

**Figure 6:**
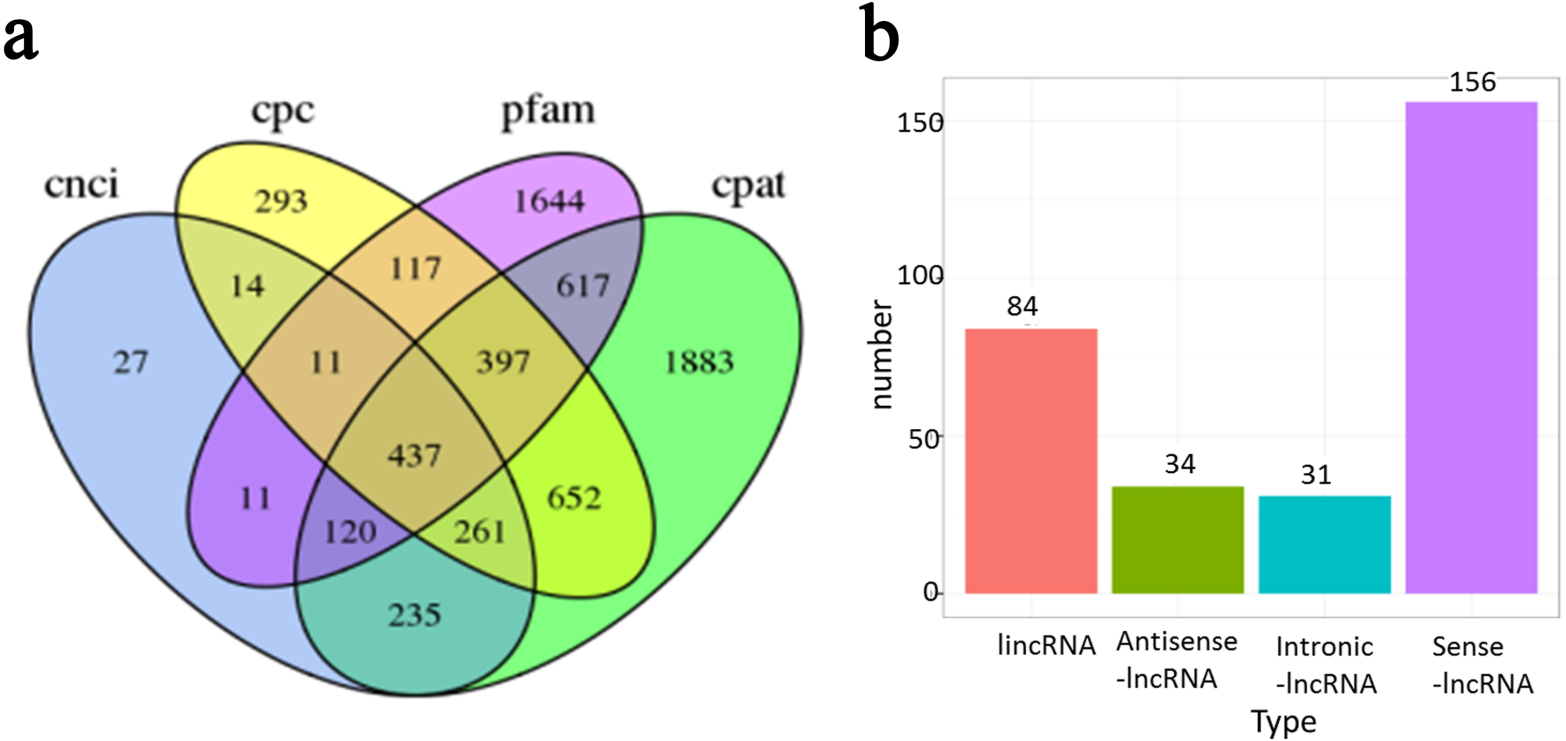
Long non-coding RNA (lncRNA) identification of *Ananas comosus* var. *bra c teatus*. (A) Venn diagram of the number of lncRNAs identified by CPC, CNCI, pfam and CPAT. (B) The distribution of lncRNAs based on their positions relative to RefGen-v3 annotations.

In addition to protein-coding RNAs, non-coding RNAs are a major component of the transcriptome. In total, 437 non-coding transcripts were identified by CPC (Kong et al., 2007), CNCI (Sun et al., 2013), Pfam and CPAT (Wang et al., 2013) analysis of the present PacBio data (Fig. 6A). Transcripts with ORFs of more than 100 amino acids were eliminated to obtain a high-confidence set of lncRNA genes. Finally, a total of 329 lncRNAs were obtained in the present study. These lncRNAs were classified into four groups based on their positions relative to RefGen-v3 annotations as follows: 51% were generated from sense strands; 28% were generated from intergenic regions; 11% were generated from antisense strands; and 10% were generated from intronic regions (Fig. 6B). Target genes were predicted for 327 of these lncRNAs and are listed in File S1.

### Fusion transcript identification

**Figure 7:**
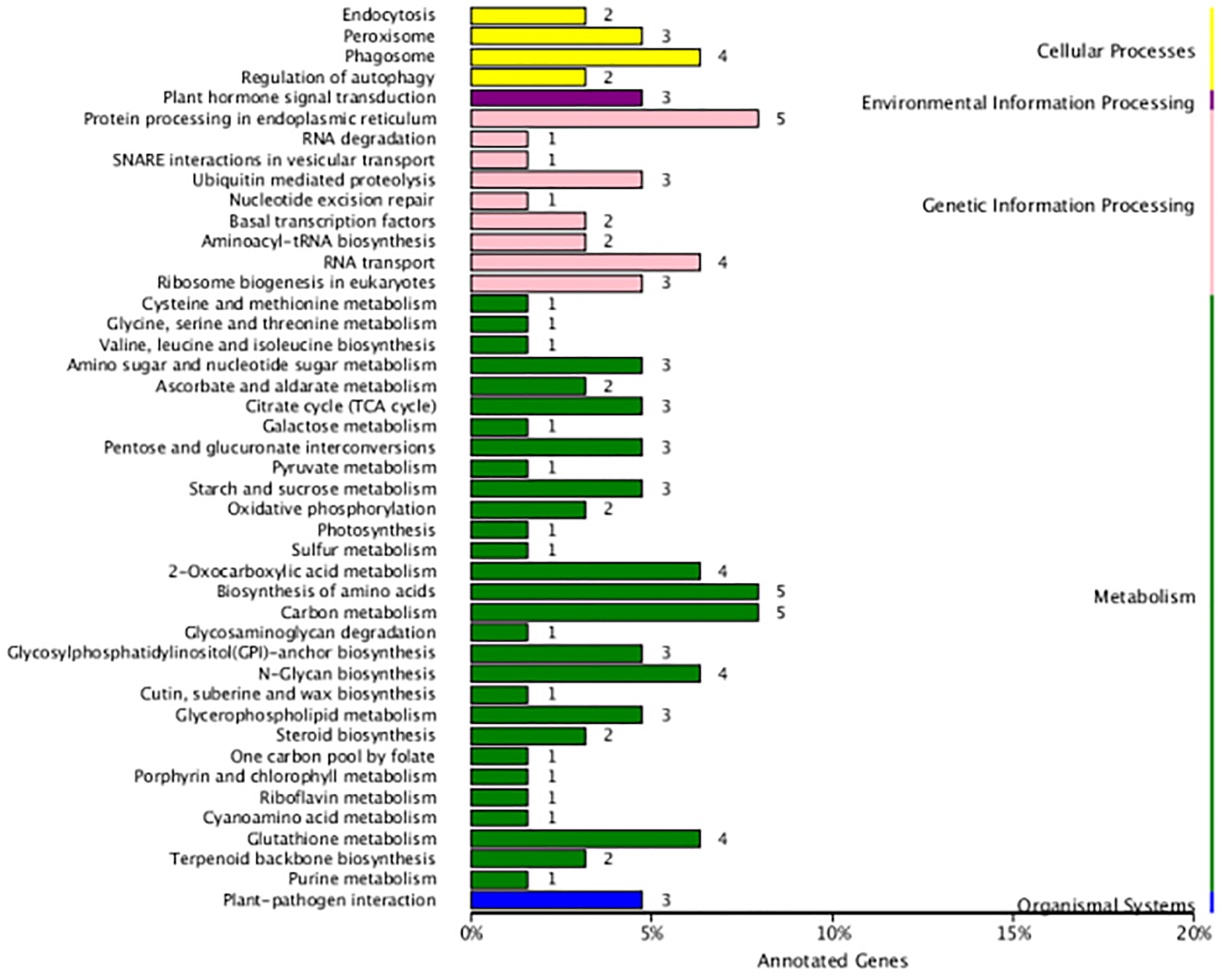
KEGG function annotation of the fusion transcripts.

In the present study, 290 fusion transcripts were identified. Similar to maize, fusion events were more likely to occur inter-chromosomally than intra-chromosomally, and most of the intra-chromosomal fusions were located on chromosomes 19, 20 and 21 (Fig. S2G). Also similar to maize (Wang et al., 2016), the sites of transcript fusion corresponded with splice junctions that also function within non-fused versions of these transcripts. It is notable that 14 fusion transcripts involved the fusion of ribulose 1,5-bisphosphate carboxylase/oxygenase large subunit N-methyltransferase with five other transcripts. Functional annotation revealed that fusion transcripts were associated with amino acid and carbon metabolism in the metabolism category as well as protein processing in the endoplasmic reticulum in the genetic information processing category (Fig. 7).

## Conclusion

Leaves, stems, and roots from 3-year-old chimeric plants as well as complete green shoots, complete white shoots, and calluses derived via tissue culture of *Ananas comosus* var. *bracteatus* were used as samples for SMRT sequencing analysis. In total, 19,838 transcripts were identified by AS analysis. Among them, 19,185 (96.7%) transcripts were functionally annotated. A total of 9,921 genes were identified by mapping the non-redundant isoforms to the reference genome. A total of 10,649 AS events were identified, the majority of which were intron retention events. There were 8,316 genes carrying at least one poly (A) site, and these genes generated 21,873 poly (A) sites in total. Moreover, 1,094 TF isoforms were identified, and 907 of these were novel isoforms. Compared to the *Acomosus* _321_v3 genome, 55 novel TFs were identified in the present data by iTAK. In the present study, 329 lncRNAs were obtained, and 290 fusion transcripts were identified. The quality of the gene structure and functional information of the obtained genome was greatly improved, which may facilitate further genetic studies of *Ananas comosus* var. *bracteatus*.

##  Supplemental Information

10.7717/peerj.7062/supp-1 Figure S1 The length distribution of each size bins (a) ROI read length distribution of each size bins. (b) FLNC read length distribution of each size bins. (c) Consensus isoforms read length distribution of each size bins.

10.7717/peerj.7062/supp-2 Figure S2 CIRCOS visualization of different data at the genome-wide level (a) Karyotype of Acomosus_321_v3 genom. (b) Gene density of genes covered by Acomosus_321_v3 genom. Gene density was calculated in a 1-Mb sliding window at 20 kb intervals. (c) Gene density of genes covered by PacBio data set. (d) Isoform density of Acomosus_321_v3 genom. Isoform density was calculated in a 1-Mb sliding window at 20 kb intervals. (e) Isoform density of PacBio data set. (f) lncRNA density of PacBio data set. (g) Linkage of fusion transcripts. Purple: intra-chromosomal; dark yellow: inter-chromosomal.

10.7717/peerj.7062/supp-3 Figure S3 Length distributions of the ORFs identified by PacBio data

10.7717/peerj.7062/supp-4 Figure S4 SSR density of different types of SSRs

10.7717/peerj.7062/supp-5 Table S1 Statistic of SSRs identified

10.7717/peerj.7062/supp-6 File S1 The target genes of the 327 lncRNAs in Ananas comosus var.*bracteatus*

10.7717/peerj.7062/supp-7 File S2 Integrated functional anotation of transcripts identified

## Additional Information and Declarations

### Data Availability

The following information was supplied regarding data availability: Data is available at GenBank, accession number: PRJNA494788.
