# Supplementary figures and images for: SMRT sequencing analysis reveals the full-length transcripts and alternative splicing patterns in Ananas comosus var. bracteatus
Source: PeerJ. 2019 Jun 21;7:e7062. doi: 10.7717/peerj.7062 (PMC6590394; doi:10.7717/peerj.7062)

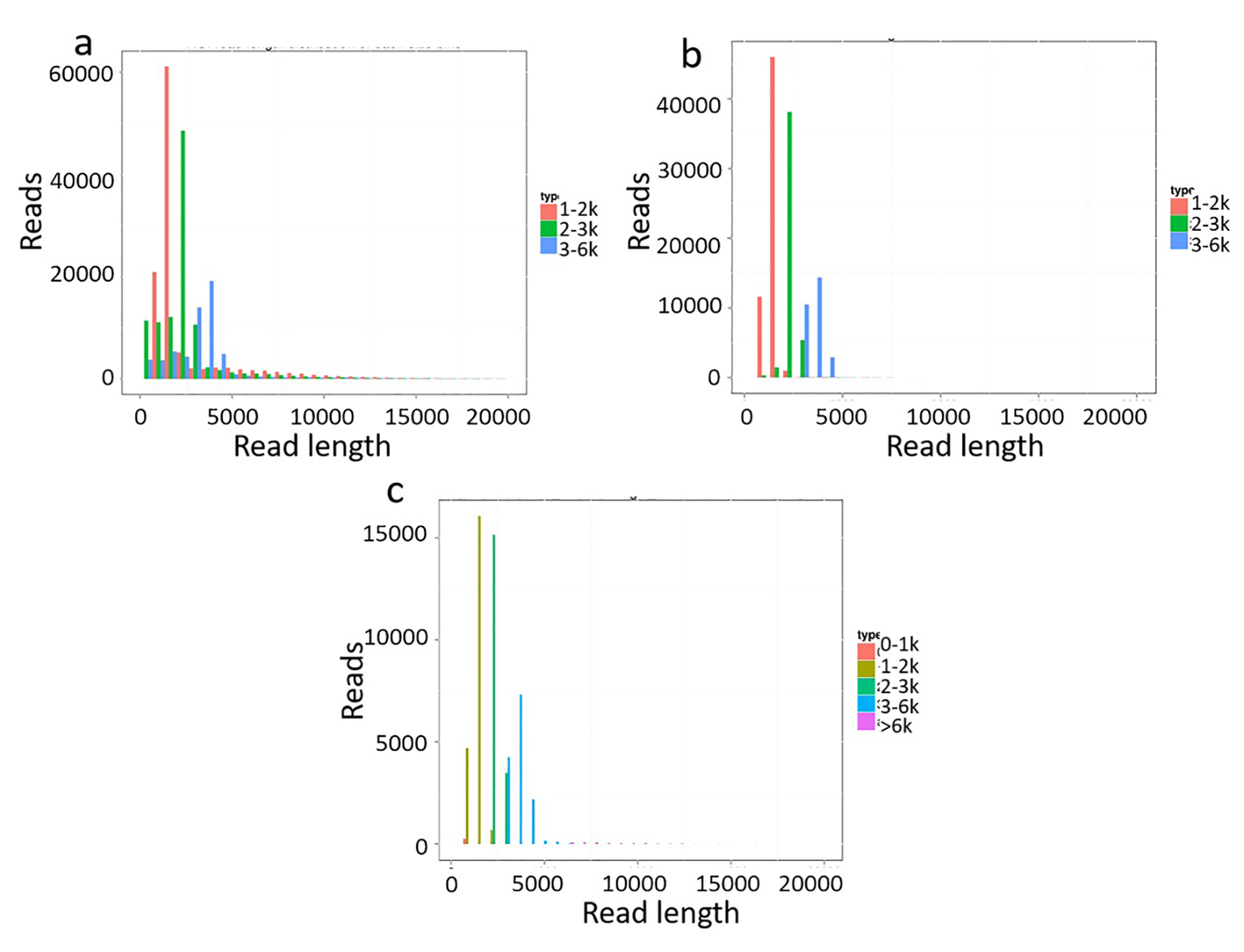

Supplement: Figure S1 — (a) ROI read length distribution of each size bins. (b) FLNC read length distribution of each size bins. (c) Consensus isoforms read length distribution of each size bins. [file peerj-07-7062-s001.png]

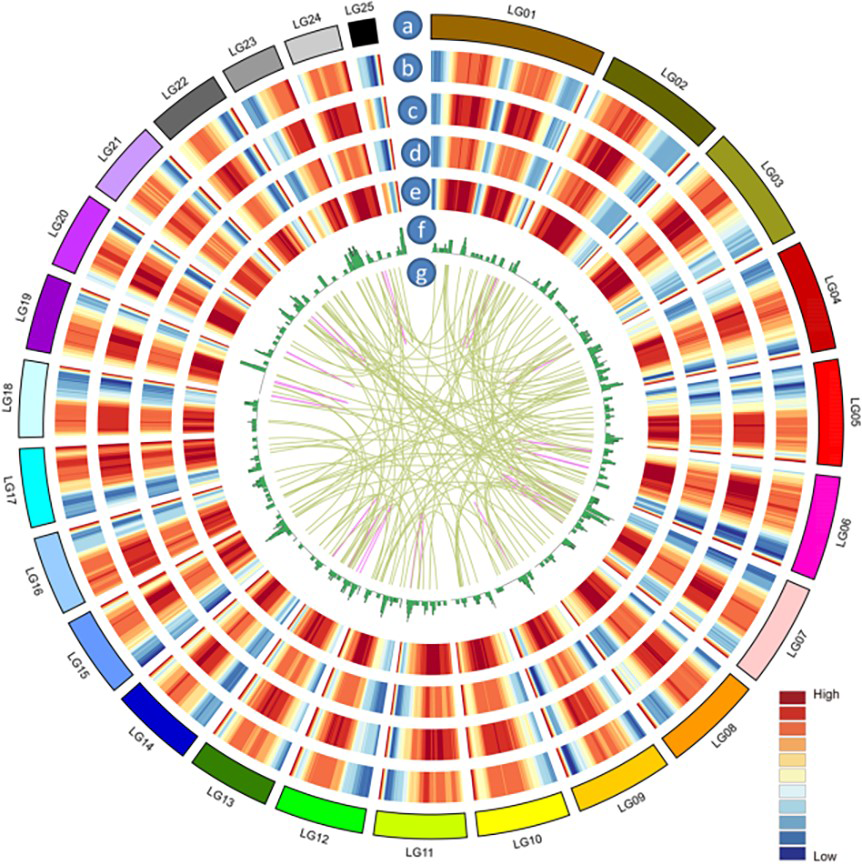

Supplement: Figure S2 — (a) Karyotype of Acomosus_321_v3 genom. (b) Gene density of genes covered by Acomosus_321_v3 genom. Gene density was calculated in a 1-Mb sliding window at 20 kb intervals. (c) Gene density of genes covered by PacBio data set. (d) Isoform density of Acomosus_321_v3 genom. Isoform density was calculated in a 1-Mb sliding window at 20 kb intervals. (e) Isoform density of PacBio data set. (f) lncRNA density of PacBio data set. (g) Linkage of fusion transcripts. Purple: intra-chromosomal; dark yellow: inter-chromosomal. [file peerj-07-7062-s002.png]

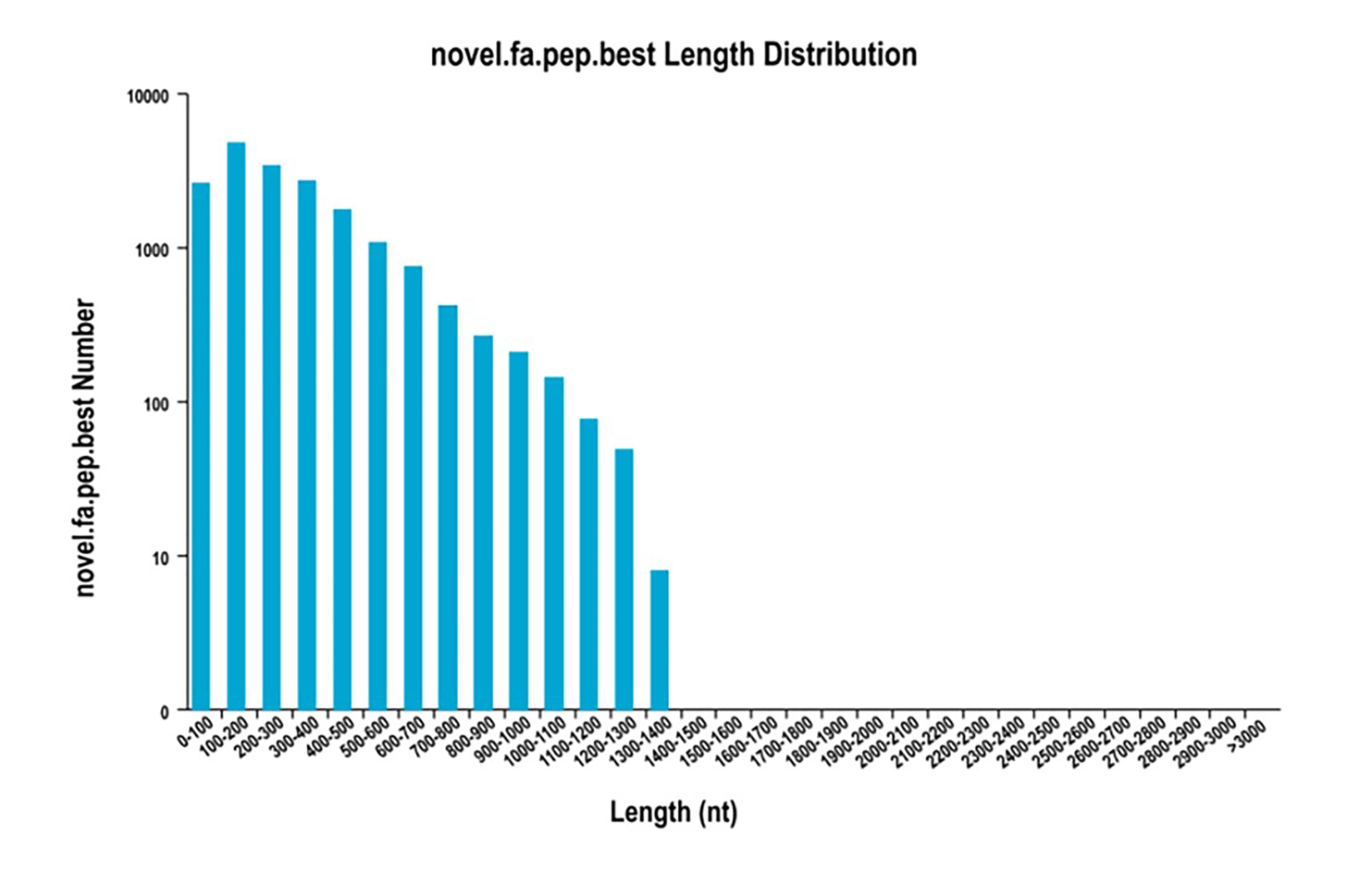

Supplement: Figure S3 [file peerj-07-7062-s003.png]

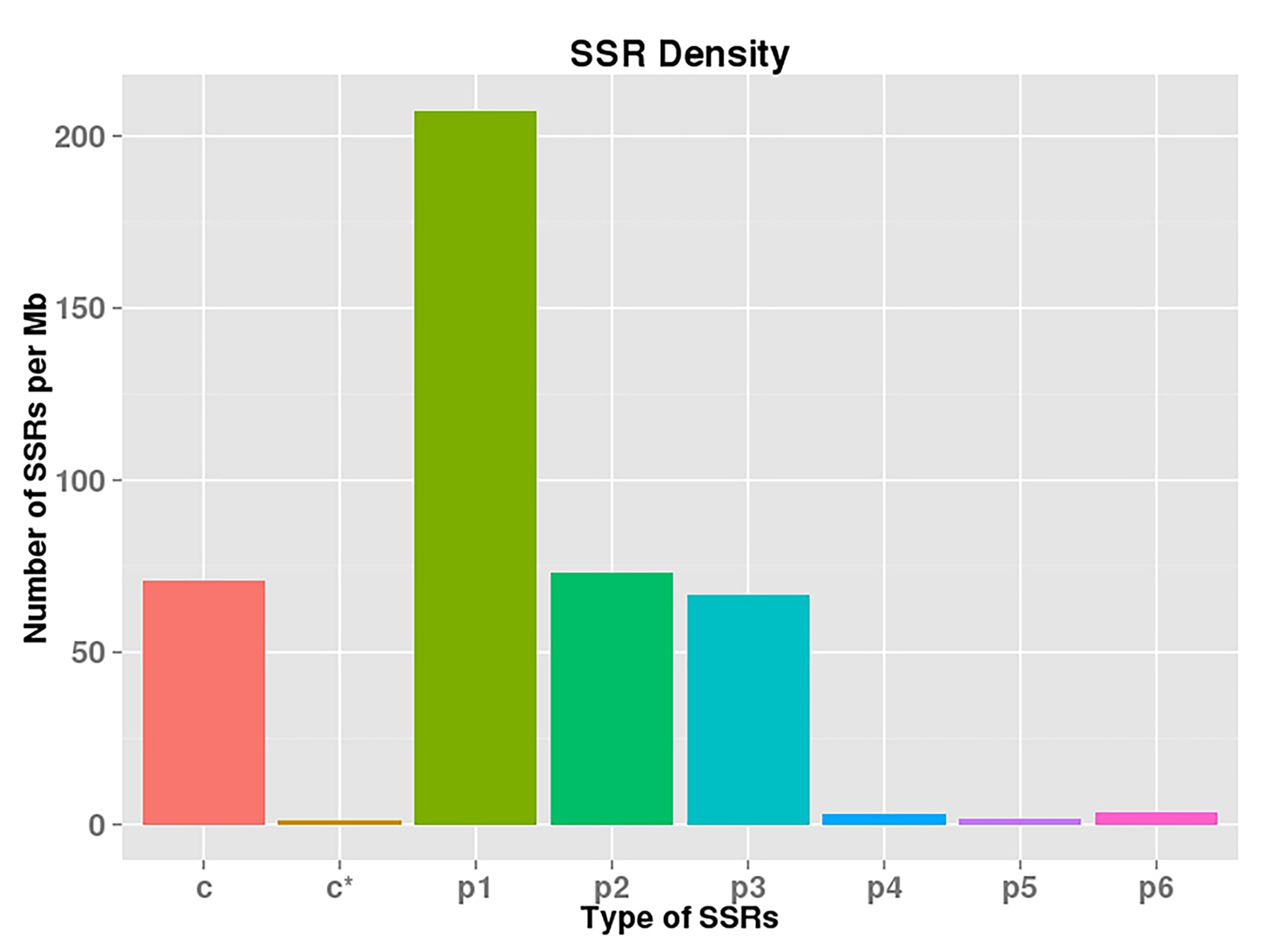

Supplement: Figure S4 [file peerj-07-7062-s004.png]
