# Supplementary material for: SMRT sequencing analysis reveals the full-length transcripts and alternative splicing patterns in Ananas comosus var. bracteatus
Source: PeerJ. 2019 Jun 21;7:e7062. doi: 10.7717/peerj.7062 (PMC6590394; doi:10.7717/peerj.7062)
Supplement: Table S1 [file peerj-07-7062-s005.docx]

**Table S1:**

**Statistic of SSRs identified.**

| Searching item | number |
| --- | --- |
| Total number of sequences examined  Total size of examined sequences (bp)  Total number of identified SSRs  Number of SSR containing sequences  Number of sequences containing more than 1 SSR  Number of SSRs present in compound formation  Mono nucleotide  Di nucleotide  Tri nucleotide  Tetra nucleotide  Penta nucleotide  Hexa nucleotide | 19,829  49,996,480  25,971  12,896  6,815  4,573  14,107  6,077  5,239  214  110  224 |
